# An improved anchor-free SAR ship detection algorithm based on brain-inspired attention mechanism

**DOI:** 10.3389/fnins.2022.1074706

**Published:** 2022-11-30

**Authors:** Hao Shi, Cheng He, Jianhao Li, Liang Chen, Yupei Wang

**Affiliations:** ^1^Radar Research Lab, School of Information and Electronics, Beijing Institute of Technology, Beijing, China; ^2^Chongqing Innovation Center, Beijing Institute of Technology, Chongqing, China; ^3^Beijing Key Laboratory of Embedded Real-Time Information Processing Technology, Beijing Institute of Technology, Beijing, China

**Keywords:** anchor-free, synthetic aperture radar, ship detection, brain-inspired, attention mechanism

## Abstract

As a computing platform that can deal with problems independently and adapt to different environments, the brain-inspired function is similar to the human brain, which can effectively make use of visual targets and their surrounding background information to make more efficient and accurate decision results. Currently synthetic aperture radar (SAR) ship target detection has an important role in military and civilian fields, but there are still great challenges in SAR ship target detection due to the problems of large span of ship scales and obvious feature differences. Therefore, this paper proposes an improved anchor-free SAR ship detection algorithm based on brain-inspired attention mechanism, which efficiently focuses on target information ignoring the interference of complex background. First of all, most target detection algorithms are based on the anchor method, which requires a large number of anchors to be defined in advance and has poor generalization capability and performance to be improved in multi-scale ship detection, so this paper adopts an anchor-free detection network to directly enumerate potential target locations to enhance algorithm robustness and improve detection performance. Secondly, in order to improve the SAR ship target feature extraction capability, a dense connection module is proposed for the deep part of the network to promote more adequate deep feature fusion. A visual attention module is proposed for the shallow part of the network to focus on the salient features of the ship target in the local area for the input SAR images and suppress the interference of the surrounding background with similar scattering characteristics. In addition, because the SAR image coherent speckle noise is similar to the edge of the ship target, this paper proposes a novel width height prediction constraint to suppress the noise scattering power effect and improve the SAR ship localization accuracy. Moreover, to prove the effectiveness of this algorithm, experiments are conducted on the SAR ship detection dataset (SSDD) and high resolution SAR images dataset (HRSID). The experimental results show that the proposed algorithm achieves the best detection performance with metrics AP of 68.2% and 62.2% on SSDD and HRSID, respectively.

## 1. Introduction

The brain-inspired concept originates from the human brain, which can focus on the target information while selectively ignoring the interference of redundant information when facing a large amount of information, and this attention mechanism in the human brain can enhance the target cognition and understanding. By imitating the processing mode of information in the human brain, the brain-inspired can improve the information acquisition ability of the target in practical applications, and finally complete the cognitive and understanding of the target.

In SAR ship detection, the target information usually contains a large number of redundant interference components, and being able to obtain the target information accurately plays an important role in the detection results. Because the brain-inspired ability to effectively pay attention to key regions in the target scene, we take SAR ship detection as an example to explore an algorithm that can effectively extract SAR ship information and improve SAR ship detection accuracy.

Synthetic aperture radar (SAR) is an active microwave imaging sensor that can effectively collect large area data under any weather conditions, such as day, night, and foggy days, and eventually generate high-resolution SAR images. Because of its all-day and all-weather high-resolution imaging capability, SAR plays an important role in marine ship target detection (Li et al., 2016), such as marine rescue, marine law enforcement and other civilian fields, as well as precise detection, ship target detection, and other military fields. However, it is difficult to detect ship targets in SAR images due to the large scale span of ship targets and obvious feature differences. Therefore, an efficient target detector is needed to detect SAR ship targets.

Traditional SAR target detection methods can be broadly classified into three categories: threshold (Wang et al., 2016), statistical (Song and Yang, 2015), and transform methods (He et al., 2019). The main steps include the pre-processing stage of processing the input image into a more recognizable image, the candidate region extraction stage of extracting possible target pixels as candidate targets, and the recognition stage of identifying targets within the potential region. Among the existing conventional SAR target detection algorithms, the constant false alarm rate (CFAR) method (Wang et al., 2017) is one of the most commonly used techniques, which is based on the main idea of establishing a sea clutter distribution model based on local sea clutter data and plotting the probability density curve of the sea clutter distribution model, then calculating the adaptive threshold based on the typical false alarm probability, and finally using the adaptive threshold to detect the target in the SAR image. Although the CFAR method has been widely used for SAR ship target detection, it relies on the modeling of sea clutter data and adapts to simple scenarios, and does not adapt to multi-scale ship detection in complex backgrounds.

With the rapid theoretical development of deep learning, various deep learning models have emerged, which are widely used in the field of image processing due to their advantages such as powerful feature characterization ability and automatic learning. For feature misalignment and variation of target appearance in SAR multi-scale target detection, Tang et al. (2022) proposed scale-aware feature pyramid network with scale-adaptive feature extraction module and learnable anchor point assignment strategy. For redundancy-oriented computation and background interference in the remote sensing domain, Deng et al. (2022) proposed fast anchor point refinement network with rotational alignment module and balanced regression loss function. To improve the SAR multi-scale ship detection performance, Cui et al. (2019) proposed dense attention pyramid network by fusing the convolutional attention module with the features of each layer to highlight the salient features of each layer. Since SAR ship targets are difficult to distinguish from the surrounding background, Yang et al. (2022) proposed robust detection network by introducing coordinate attention approach to obtain more representative semantic features. To obtain better detection performance in practical industrial applications, Gao et al. (2022) proposed efficient SAR ship detection network with targeted skill fusion strategy based on Yolov4.

The above detection algorithms are all based on anchor detectors, and although these methods achieve better performance in target detection, there are still some shortcomings. Firstly, the algorithms need to manually set some hyperparameters according to the data, which are sensitive to ship targets with large scale span. Secondly, the algorithms usually generate a large number of anchor boxes on the image, while SAR ship targets account for a small percentage of the image, and a large number of irrelevant anchor boxes waste computational resources. Moreover, when the targets are densely arranged, the overlapping area of candidate anchor boxes is large, and some targets are missed under non-maximum suppression. Therefore, it is necessary to propose an efficient anchor-free detector in SAR ship target detection.

The general anchor-free detector is designed based on natural scene images, while SAR images are very different from natural scene images, and the detection results are not good if the anchor-free detector is directly applied to SAR ship target detection. First of all, the SAR image coherent speckle noise is relatively large, the ship target is relatively similar to the clutter, and the island, port and building backgrounds have high grayscale characteristics easily confused with the ship target, so the SAR ship target features are difficult to extract, and problems such as missed detection and false detection are easy to occur in the detection results. In order to detect objects more effectively in existing detection algorithms, Deng et al. (2021) introduced dynamic weights to encourage the filters to focus on more reliable regions during the training phase. Han et al. (2019) added global context patches in the training phase of the model to better distinguish the target from the background. Zhao et al. (2017) adopted high confidence update strategies and study mechanisms to avoid model corruption and handle occlusion. Han et al. (2017) utilized a co-training paradigm to formulate multi-feature templates with inherently complementary information into a correlation filter model to extract valid feature targets. Wang et al. (2022) introduced deep residual networks into dictionary learning to extract rich image information. Lin et al. (2017a) developed top-down architectures with lateral connections for building high-level feature maps at various scales. Although existing feature extraction networks can effectively extract target features, they often lack the targeting of different feature layers in the network. The deep part of the network has a relatively large perceptual field and rich semantic features, and we propose a dense connection module for the deep part of the network to promote more adequate deep feature fusion. The shallow part of the network has a relatively small perceptual field and rich fine-grained details, and we propose a visual attention module for the shallow part of the network to focus on the salient features of the ship target in the local area for the input SAR images and suppress the interference of the surrounding background with similar scattering characteristics. In addition, because the scattered power distribution of the surrounding background in the near-shore scene of SAR images is similar to the edge of the ship target, it is easy to lead to the offset between some ship predicted positions and real positions, and the ship target is not localized correctly. For this reason, we propose a novel width height prediction constraint, which considers the overlapping area of the predicted box and the real box, the real difference between the width and length of the edge and the loss gradient reweighting to improve the ship target localization accuracy.

In conclusion, drawing on the idea that the brain-inspired can effectively use visual targets and their surrounding background information, we propose an improved anchor-free SAR ship detection algorithm based on brain-inspired attention mechanism. The main contributions are summarized as follows.

1. We propose an improved anchor-free SAR ship detection algorithm, which directly enumerates potential target locations and classifies them with better generalization capability compared to the anchor method, and makes targeted improvements to different feature layers of the network to improve SAR ship detection accuracy.
2. We design a dense connection module and a visual attention module for feature extraction. The deep part of the network is richer in semantic features, and the dense connection module promotes more adequate deep feature fusion. The shallow part of the network is richer in fine-grained details, and the visual attention module focuses on the salient features of the target in the local area and suppresses the surrounding background interference, which can eventually detect the SAR ship target more effectively.
3. We design a novel width height prediction constraint, which considers the overlapping area of the prediction box and the real box, the real difference between the length and width of the edge and the loss gradient reweighting, which suppresses the influence of the near-shore background on SAR ship target localization and improves the SAR ship target localization accuracy.

## 2. Related work

Since the concept of deep learning (Hinton and Salakhutdinov, 2006) was proposed, deep learning has gradually shown great advantages over traditional methods for various classification and regression tasks, and target detection using deep learning has now become mainstream. Existing target detection methods are mainly divided into two categories: anchor-based detectors and anchor-free detectors.

In the anchor-based detectors, first a series of sliding windows are predefined on the feature map, then they are divided into positive and negative samples according to the IOU values, and finally the detection results are obtained by classification regression on the divided positive and negative samples. The anchor-based detectors can be classified into two-stage and one-stage detectors according to the number of classification regression. Typical representatives of two-stage detectors are Faster R-CNN (Ren et al., 2017), Cascade R-CNN (Cai and Vasconcelos, 2018), etc., while typical representatives of one-stage detectors are RetinaNet (Lin et al., 2017b), SSD (Liu et al., 2016), etc. Generally speaking, two-stage detectors can obtain higher accuracy, but the processing speed is slower. One-stage detectors have faster processing speed, but obtain poorer accuracy.

Anchor-based detectors require a series of predefined sliding windows before detecting targets, while brain-inspired detects important area targets directly without predefined operations. In addition, the predefined sliding windows are not suitable for the targets with large scale span in remote sensing image processing, so the anchor-free detectors have been developed and researched.

In the anchor-free detectors, they can be mainly divided into key point detectors and pixel point detectors. In this paper, we focus on key point detectors, which detect the key points of the same instance object after prediction by identifying the location of bounding box characteristics as key points. The typical representatives of key point detectors are CornerNet (Law and Deng, 2018), ExtremeNet (Zhou et al., 2019b), and CenterNet (Zhou et al., 2019a), etc. CornerNet predicts the top-left and bottom-right points of the target and determines the connection between the two points through the localization vector to complete the target detection, but when the target is irregular, the extracted information of the two points is weak. ExtremeNet predicts the center point of the target and the extreme points of the four edges of the target to complete the target detection, but the network outputs a large number of key points and requires a large number of extreme points to be matched, resulting in a slow operation. Based on the above methods, Centernet determines the target location directly by predicting the center of the target without subsequent grouping and post-processing, and the network will be described in detail later. Although anchor-based detectors dominate in target detection, the anchor-free detectors processing idea is more scientific and have great potential for development.

## 3. Methods

**Figure 1:**
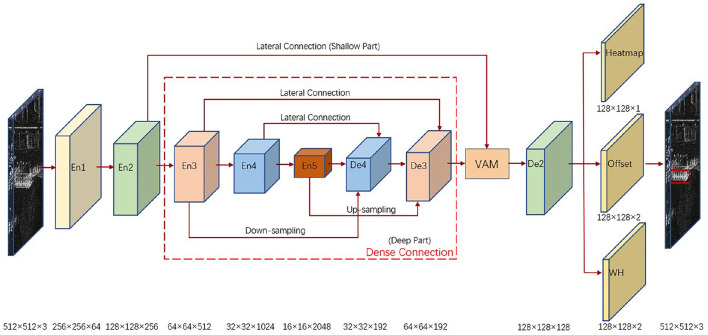
Overall architecture of the proposed algorithm. The deep part of the network is densely connected, the shallow part of the network is processed by the visual attention module (VAM), and the prediction head outputs heatmap, target center offset, and constrained target width and height.

The overall architecture of our proposed algorithm is shown in Figure 1, using an anchor-free network with an encoder-decoder structure, which performs targeted feature extraction for the deep and shallow parts of the network with target width and height prediction constraint to finally obtain detection results. In the deep part of the network, a dense connection module is made from the encoder layer En3 to the decoder layer De3 to promote a more adequate deep feature fusion. In the shallow part of the network, the encoder layer En2 is processed with a visual attention module to focus on the salient features of the local area ship targets for the input SAR images and suppress the interference of the surrounding background with similar scattering characteristics. In the prediction head part of the network, the decoder layer De2 outputs heatmap, target center offset, and constrained target width and height to obtain the final detection results.

In this section, we first introduce the anchor-free network with an encoder-decoder structure used as the algorithm baseline. Next, the designed dense connection module and visual attention module are described in detail. Then we present a novel width height prediction constraint designed in the prediction head.

### 3.1. Anchor-free network

The proposed algorithm builds on the key point anchor-free detector, which determines the target center by key point estimation and regresses at the target center to obtain other target attributes, such as target center offset and target width and height.

The feature extraction part uses an encoder-decoder structure. In the encoder, Resnet101 is used for feature extraction, and the extracted features are En1, En2, En3, En4, En5, with scales corresponding to 1/2, 1/4, 1/8, 1/16, 1/32 of the original image, reflecting the information of SAR image from shallow to deep. The shallow features are richer in fine-grained details and highlight the boundary of the target, while the deep features are richer in semantic features and highlight the location of the target. In the decoder, the features extracted by the encoder are up-sampled three times to gradually recover the feature map resolution, and the up-sampled features are De4, De3, De2, with scales corresponding to 1/16, 1/8, 1/4 of the original image. The final network output features are not only rich in feature extraction, but also have higher resolution, which is convenient for target detection.

In the prediction head part of the network, the output heatmap, target center offset and target width and height are shown in Figure 1. Heatmap is used to locate the key points to be determined in the input image, and the peak in the heatmap is determined as the center of the target by sigmoid function processing. Since the spatial resolution of the output heatmap is 1/4 of the original image, the target center offset is used to compensate for the pixel error caused by mapping the points on the heatmap to the original image. The output target width and height is used to predict the size of the target. Compared with the anchor detector, the key point anchor-free detector directly predicts the target center to determine the target, which is more in line with the idea of brain-inspired attention mechanism.

### 3.2. Dense connection module

**Figure 2:**
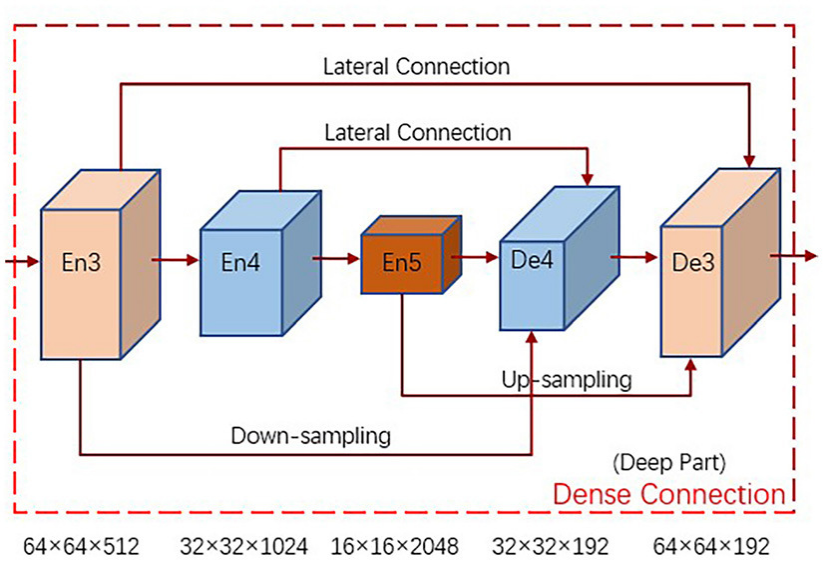
The dense connection module is used for feature extraction, which can promote the deep feature fusion more fully.

We design a dense connection module for feature extraction to promote more adequate deep feature fusion. Traditional feature extraction methods usually utilize lateral connection to combine high-level semantic feature mappings from the decoder with corresponding low-level detailed feature mappings from the encoder, which can extract effective target features but lack correlation between adjacent layers and feature extraction is not sufficient. For this reason, we design a dense connection module, as shown in Figure 2, with decoder feature layers from the encoder small-scale and same-scale feature mappings, and large-scale feature mappings from the decoder or encoder layer En5, to promote adequate feature fusion.

The encoder layer En1 is usually not considered in the following feature extraction, while the shallow part of the network En2 is not sufficiently extracted with still more background interference, so we only process the deep part of the network, i.e., the dense connection from the encoder layer En3 to the decoder layer De3, to promote the deep feature fusion more fully. Take how to build the decoder layer De4 as an example, its input sources are, the encoder layer En3 after down-sampling operation, the encoder layer En4 after lateral connection and the encoder layer En5 after up-sampling operation, whose feature maps have the same resolution for channel concatenation, and the number of channels of each input feature layer is 64 in order to unify the number of channels. To fuse the concatenated feature maps more fully, a fusion process is applied to them, i.e., a convolution of size 3 × 3 with 192 channels, batch normalization and ReLU activation function. The formula for constructing the decoder layer De4 is as follows:

(1) $$\begin{array}{l} De4=FP\left(CONCAT\left(D\left(En3\right),L\left(En4\right),U\left(En5\right)\right)\right) \end{array}$$

where D(·) denotes the down-sampling operation, L(·) denotes the lateral connection, U(·) denotes the up-sampling operation, CONCAT(·) performs channel concatenation on the three processed feature maps, and FP(·) applies fusion processing on the concatenated feature maps by convolution, batch normalization with RELU activation function.

### 3.3. Visual attention module

**Figure 3:**
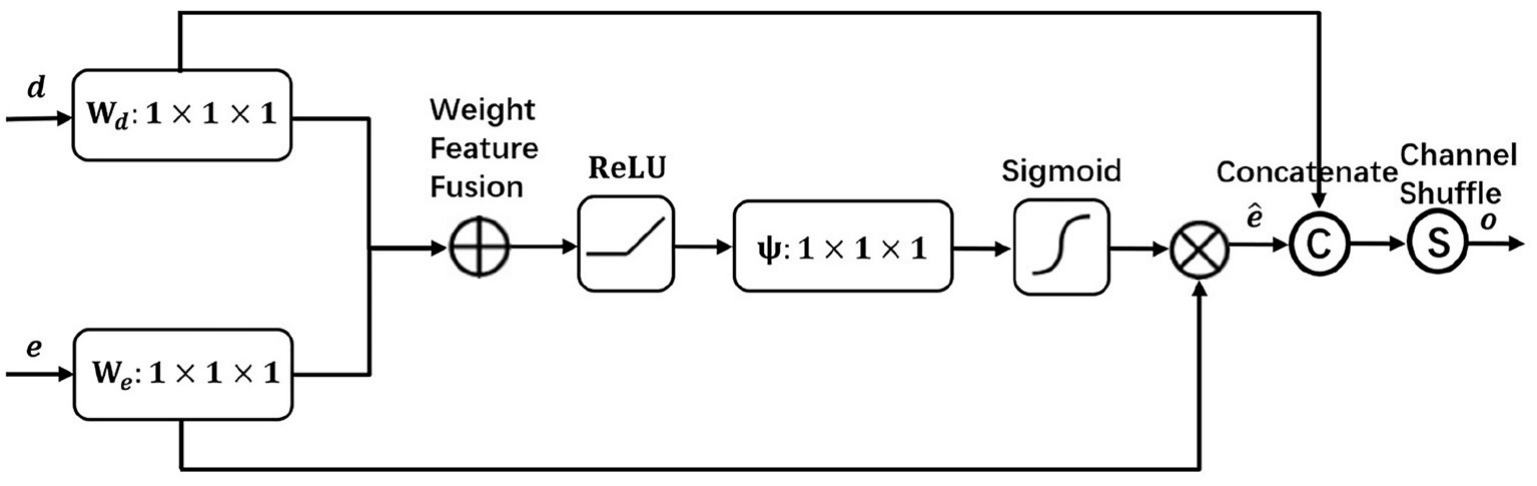
The visual attention module focuses on the salient features of the target in the local area, suppresses the surrounding background interference, and detects the object effectively.

We design a visual attention module to focus on local area SAR ship target salient features, suppress surrounding background interference, and finally detect the target effectively. As shown in Figure 3, encoder feature e and decoder feature d are input to the network for attention processing to obtain encoder feature $\hat{e}$ that highlights important information, and the processed encoder feature $\hat{e}$ is then channel concatenated and shuffled with decoder feature d, thus promoting sufficient information mixing among different channels and finally obtaining feature o for target detection.

In the shallow part of the network, the encoder layer En2 is rich in fine-grained details, but it is usually ignored in the feature extraction due to insufficient feature extraction and still more background interference. To extract richer features in SAR target detection, we apply the visual attention module to the encoder layer En2 and the decoder layer De3 with the same scale after up-sampling, so as to obtain the effective feature information of the encoder layer En2 and finally achieve better detection results. In the visual attention module, the encoder layer En2 is simplified as feature e and the processed decoder layer De3 is simplified as feature d. First, they go through a 1 × 1 convolution *W*_*e*_ and *W*_*d*_, respectively to change the channels into the same, followed by a weigh feature fusion of both, i.e., a selective element-by-element summation with differentiated fusion of different input features, and then after a Relu activation function, a 1 × 1 convolution ψ of the channel down to 1 and Sigmoid to obtain the attention coefficients. By using the attention coefficients to weight the encoder features e, the encoder features $\hat{e}$ that highlight the effective information are obtained, and the processed encoder features $\hat{e}$ are channel concatenated and shuffled with the decoder features d, thus promoting information mixing among different channels and finally obtaining feature o for target detection. The visual attention module is processed by the following equation:

(2) $$\begin{array}{l} WFF=Conv\left(\frac{\omega_{1}\times I_{1}+\omega_{2}\times I_{2}}{\omega_{1}+\omega_{2}+\varepsilon }\right) \end{array}$$

(3) $$\begin{array}{l} \hat{e}=SIG\left(\text{Ψ}\left(RELU\left(WFF\left(e\times W_{e},d\times W_{d}\right)\right)\right)\right)\times \left(e\times W_{e}\right) \end{array}$$

(4) $$\begin{array}{l} o=CS\left(CONCAT\left(\hat{e},d\times W_{d}\right)\right) \end{array}$$

In (2), WFF represents weight feature fusion, where w is the parameter we learn to distinguish the importance of different input features I in the feature fusion process. In (3), $\hat{e}$ represents the encoder features with salient important information, where SIG(·) denotes the sigmoid function. In (4), o represents the features processed by the visual attention module for target detection, where CS(·) denotes channel shuffle and CONCAT(·) denotes channel concatenation.

### 3.4. Width height prediction constraint

**Figure 4:**
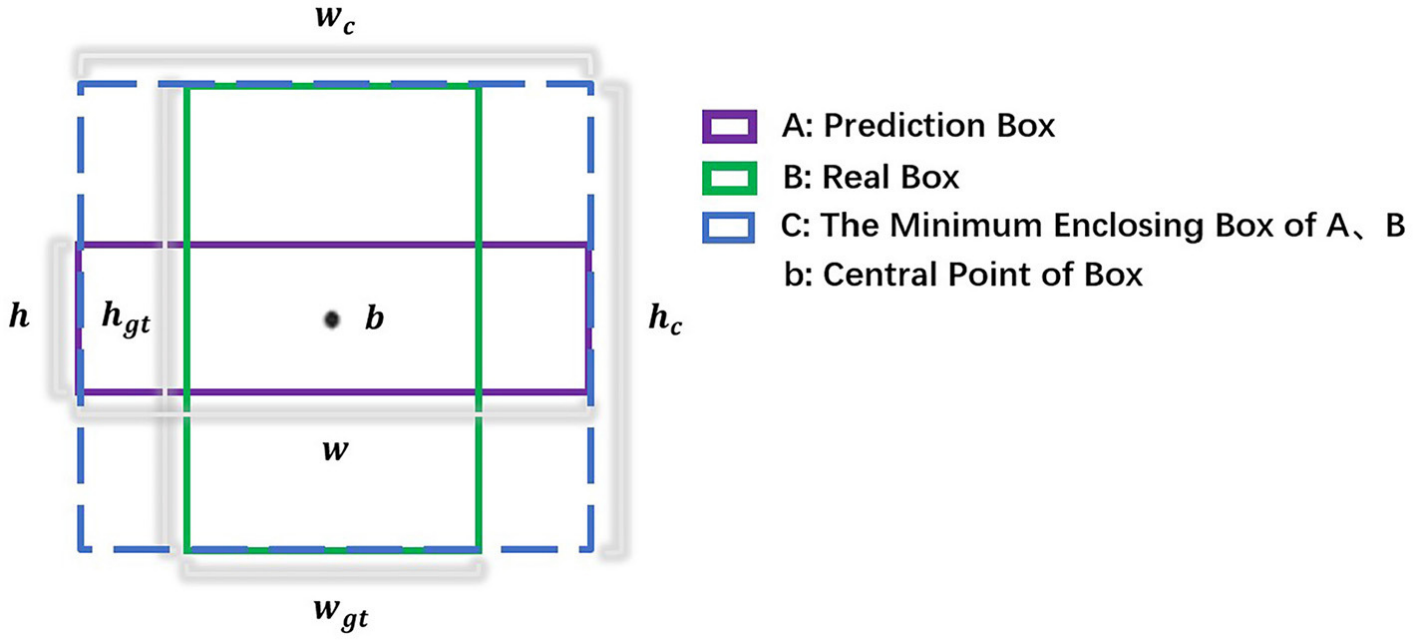
The relative position of the prediction box and the real box, both of which have the same center.

In predicting the width and height of the target, the scattered power distribution of the surrounding background in the near-shore scene of SAR image is relatively similar to the edge of the ship target, which is easy to have an impact on the ship target localization. So we propose a new width height prediction constraint, considering the overlapping area of the prediction box and the real box, the real difference of width and height edge and the loss gradient reweighting to improve the ship target localization accuracy. The relative position of the prediction box and the real box is shown in Figure 4. In wide and high prediction, the network only computes the positive sample loss values, so the prediction box overlaps with the true box at the center.

The overlapping area of prediction box and real box is better for ship targets with large scale differences, which can make the width and height regressions have the same contribution at different scales. The true difference of width and height edge can minimize the difference between the width and height of the prediction box and the true box to improve the detection accuracy. Loss gradient reweighting is better when focusing on high IOU targets by adaptively enhancing the weighting of the loss and gradient of high IOU objects. The width height prediction constraint loss function is as follows:

(5) $$\begin{array}{l} L_{\text{si}ze}=1-IOU^{\alpha }\left(A,B\right)+\frac{\rho^{2\alpha }\left(w,w_{gt}\right)}{{w_{c}}^{2\alpha }}+\frac{\rho^{2\alpha }\left(h,h_{gt}\right)}{{h_{c}}^{2\alpha }} \end{array}$$

where *L*_si*ze*_ represents the width height prediction constraint loss value, *IOU*(*A, B*) considers the overlapping area of the prediction box and the real box, $\frac{\rho^{2}\left(w,w_{gt}\right)}{{w_{c}}^{2}}+\frac{\rho^{2}\left(h,h_{gt}\right)}{{h_{c}}^{2}}$ considers the real difference of width and height edge, *w*_*c*_ and *h*_*c*_ denote the width and height of the minimum external box covering the prediction box and the real box, and α considers the loss gradient reweighting, by adjusting the parameter α, the detector can flexibly achieve different IOU target regression accuracy, the parameter α is taken as 3.

## 4. Experiments

To evaluate the performance of the proposed algorithm, we conducted experiments on the SAR ship detection dataset (SSDD) and high resolution SAR images dataset (HRSID). Firstly, the adopted dataset, experimental setup and evaluation metric are described. Then ablation experiments are performed on the algorithm to verify the effectiveness of the proposed dense connection module, visual attention module, and width height prediction constraint. Finally, it is compared with multiple target detection methods to demonstrate that the proposed algorithm can achieve better results in SAR ship target detection.

### 4.1. Implementations

#### 4.1.1. Dataset

SSDD is the first publicly available dataset at home and abroad dedicated to SAR image ship target detection, which can be used for training and testing to check algorithms and is widely used. SSDD contains a total of 1,160 images, each image size is about 500 × 500, with a total of 2,456 ships, and the average number of ships per image is 2.12. The data mainly has RadarSat-2, TerraSAR-X and Sentinel-1 sensors with four polarizations of HH, HV, VV, and VH, and resolutions of 1–15 m, with ship targets in large areas of the sea and nearshore. We choose the suffix images with indexes 1 and 9 as the test set (232 images). The images with index suffix 7 are set as the validation set (116 images). The remaining images in SSDD are set as the training set (812 images). The image size is resized to 512 × 512 in our experiment.

HRSID is a high-resolution SAR ship detection dataset that includes SAR images of different resolutions, polarization, sea state, sea area, and coastal ports. The dataset is collected by Sentinel-1 and TerraSAR-X satellites and contains a total of 5,604 high-resolution SAR images and 16,951 labeled ship targets. Based on the original report in the HRSID dataset, the whole dataset is divided into training and test sets according to 13:7. The image size is 800 × 800 in our experiment.

#### 4.1.2. Experimental setup

The proposed algorithm is implemented on pytorch 1.4.0, CUDA 10.1, and NVIDIA TITAN RTX GPU. Adam is used to optimize the target, the initial learning rate is 1.25e-4, the batch size is 16, and the feature extraction backbone is Resnet-101.

#### 4.1.3. Evaluation metric

To evaluate the algorithm performance, we use the COCO metrics, which are AP, *AP*_50_, *AP*_75_, *AP*_*s*_, *AP*_*m*_, and *AP*_*l*_. The average precision (mAP) is the area under the precision–recall curve, which reflects the average precision of multiple types of targets. mAP = AP since there is only one type of target for SAR ships. The IoU threshold is calculated every 0.05 on the interval from 0.5 to 0.95, and the final average is taken as the final result of AP. *AP*_50_ is the AP at IOU = 0.5 and *AP*_75_ is the AP at IOU = 0.75. *AP*_75_ requires more stricter target localization accuracy. *AP*_*s*_, *AP*_*m*_ and *AP*_*l*_ correspond to the AP of small-scale, medium-scale and large-scale targets, respectively. The precision and recall equations are as follows:

(6) $$\begin{array}{l} Precision=\frac{TP}{TP+FP} \end{array}$$

(7) $$\begin{array}{l} Recall=\frac{TP}{TP+FN} \end{array}$$

where TP is the number of correctly detected ships, FP is the number of false alarm ships, FN is the number of missed ships.

### 4.2. Ablation experiments

We performed ablation experiments on SSDD to analyze the contribution of the proposed different modules. To ensure the validity of the experimental results, all experimental settings are the same. The results are shown in Table 1, and it can be seen that the proposed different modules all significantly improve the algorithm and enhance the SAR ship target detection accuracy.

**Table 1:** Contribution of dense connection module, visual attention module, and width height prediction constraint to the algorithm on SSDD.

**Dense connection**	**Visual attention**	**Prediction constraint**	**AP**	***AP*_50_**	***AP*_75_**	***AP*_*s*_**	***AP*_*m*_**	***AP*_*l*_**
×	×	×	0.605	0.938	0.723	0.565	0.667	0.673
√	×	×	0.665	0.964	0.804	0.632	0.715	0.733
×	√	×	0.622	0.965	0.734	0.582	0.679	**0.746**
×	×	√	0.629	0.942	0.750	0.594	0.680	0.712
√	×	√	0.672	0.965	0.814	**0.649**	0.707	0.716
√	√	√	**0.682**	**0.968**	**0.817**	0.647	**0.736**	0.717

Bold values indicate that the value is the largest in the same metric.

In the experimental results, by comparing the results in the second row with the fifth row, the combination of the dense connection module with the width height prediction constraint improves more in the metrics *AP*_*s*_ and *AP*_75_, with an increase of 1.7 and 1.0%, respectively. *AP*_*s*_ indicates the extraction ability of small-scale ships, and *AP*_75_ requires high target localization accuracy, which we believe is mainly due to the consideration of the overlapping area between the prediction box and the real box in the width-height prediction, and the introduction of the loss gradient reweighting. The overlapping area makes the target width and height regressions have the same contribution at different scales, which avoids the network from focusing too much on large scale ships and ignoring the importance of small scale ships. The loss gradient reweighting improves the loss of high IOU and improves the target localization accuracy. By comparing the fifth row with the last row of results, the combination of adding the visual attention module improves more in the metric *AP*_*m*_, which is 2.9% higher than before. The dense connection module acts on deep features with relatively large sensory fields, which usually correspond to the extraction of medium and large scale targets, and we believe that the visual attention module adds shallow detail information to the deep extracted features, which enriches the network features and promotes the target detection accuracy.

**Figure 5:**
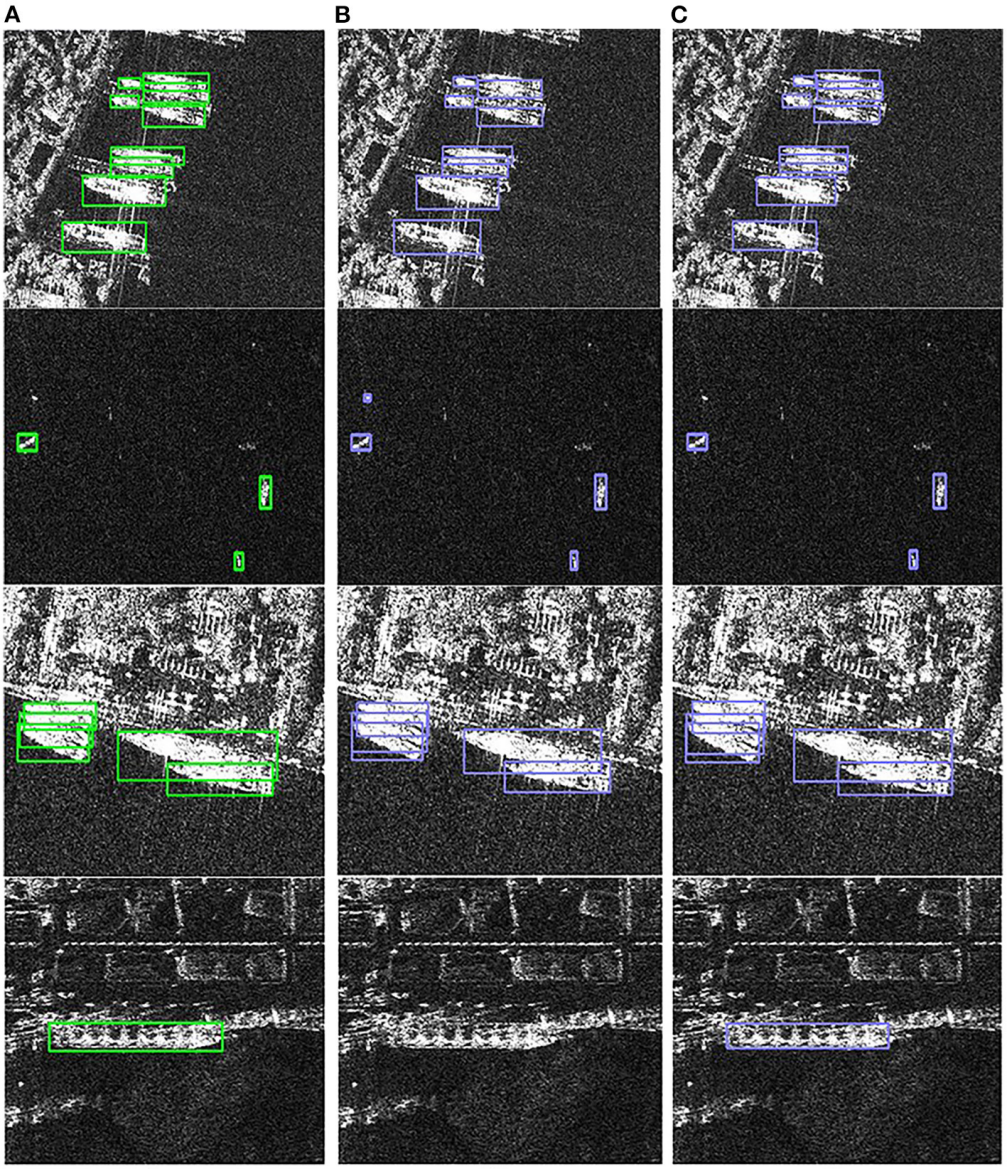
Detection results of different proposed modules. **(A)** Ground truth. **(B)** Detection results without proposed modules. **(C)** Detection results with proposed modules.

The detection results of the different modules proposed are shown in Figure 5. The first row (Figures 5B,C) shows the detection results without and with the dense connection module, respectively. The dense connection module can detect the missed ship target and improve the target detection accuracy. The second and third rows (Figures 5B,C) show the detection results without and with the width height prediction constraint, respectively. The results in the second row show that the width height prediction constraint can avoid the small target with false alarm and improve the small target detection accuracy. The results in the third row show that the width height prediction constraint makes the ship's tail localization more accurate and improves the target localization accuracy. The last row (Figures 5B,C) shows the detection results without and with visual attention module, respectively, and the visual attention module reduces the interference of near-shore background and improves the target detection accuracy.

### 4.3. Performance and analysis

In order to verify the effectiveness of this algorithm in SAR ship target detection, this algorithm is compared with multiple target detection methods. The feature extraction backbone is used Resnet101 and keeps other parameters consistent. The AP metric can reflect the overall performance of target detection, and according to Table 2, the proposed algorithm achieves 68.2% AP on SSDD, which is 3.7, 5.4, 4.2, 2.6, and 1.2% higher than Faster R-CNN, RetinaNet, FCOS, ATSS, and VFNet, respectively, which proves the effectiveness of the proposed algorithm on SAR ship target detection. In addition, the proposed algorithm has higher detection accuracy than other methods except in the metric *AP*_*m*_ which is lower than VFNet, and metric *AP*_*l*_ which is lower than Faster R-CNN. According to Table 3, the proposed algorithm achieves 62.2% AP on HRSID, and the AP, *AP*_50_, *AP*_75_, *AP*_*s*_, *AP*_*m*_, and *AP*_*l*_ are 5.4, 3.2, 7.9, 6.2, 1, and 0.7% higher than those evaluated on baseline, respectively, which proves the robustness of the proposed algorithm on different datasets.

**Table 2:** Performance of other target detection methods and the proposed algorithm on SSDD.

**Method**	**AP**	***AP*_50_**	***AP*_75_**	***AP*_*s*_**	***AP*_*m*_**	***AP*_*l*_**
Faster R-CNN	0.645	0.925	0.774	0.592	0.724	**0.793**
RetinaNet	0.628	0.943	0.741	0.568	0.726	0.661
FCOS	0.640	0.940	0.758	0.598	0.714	0.691
ATSS	0.656	0.958	0.770	0.603	0.741	0.744
VFNet	0.670	0.965	0.802	0.622	**0.746**	0.737
Proposed	**0.682**	**0.968**	**0.817**	**0.647**	0.736	0.717

Bold values indicate that the value is the largest in the same metric.

**Table 3:** Performance of the baseline method and the proposed algorithm on HRSID.

**Method**	**AP**	***AP*_50_**	***AP*_75_**	***AP*_*s*_**	***AP*_*m*_**	***AP*_*l*_**
Baseline	0.568	0.866	0.619	0.567	0.679	0.347
Proposed	**0.622**	**0.898**	**0.698**	**0.629**	**0.689**	**0.354**

Bold values indicate that the value is the largest in the same metric.

**Figure 6:**
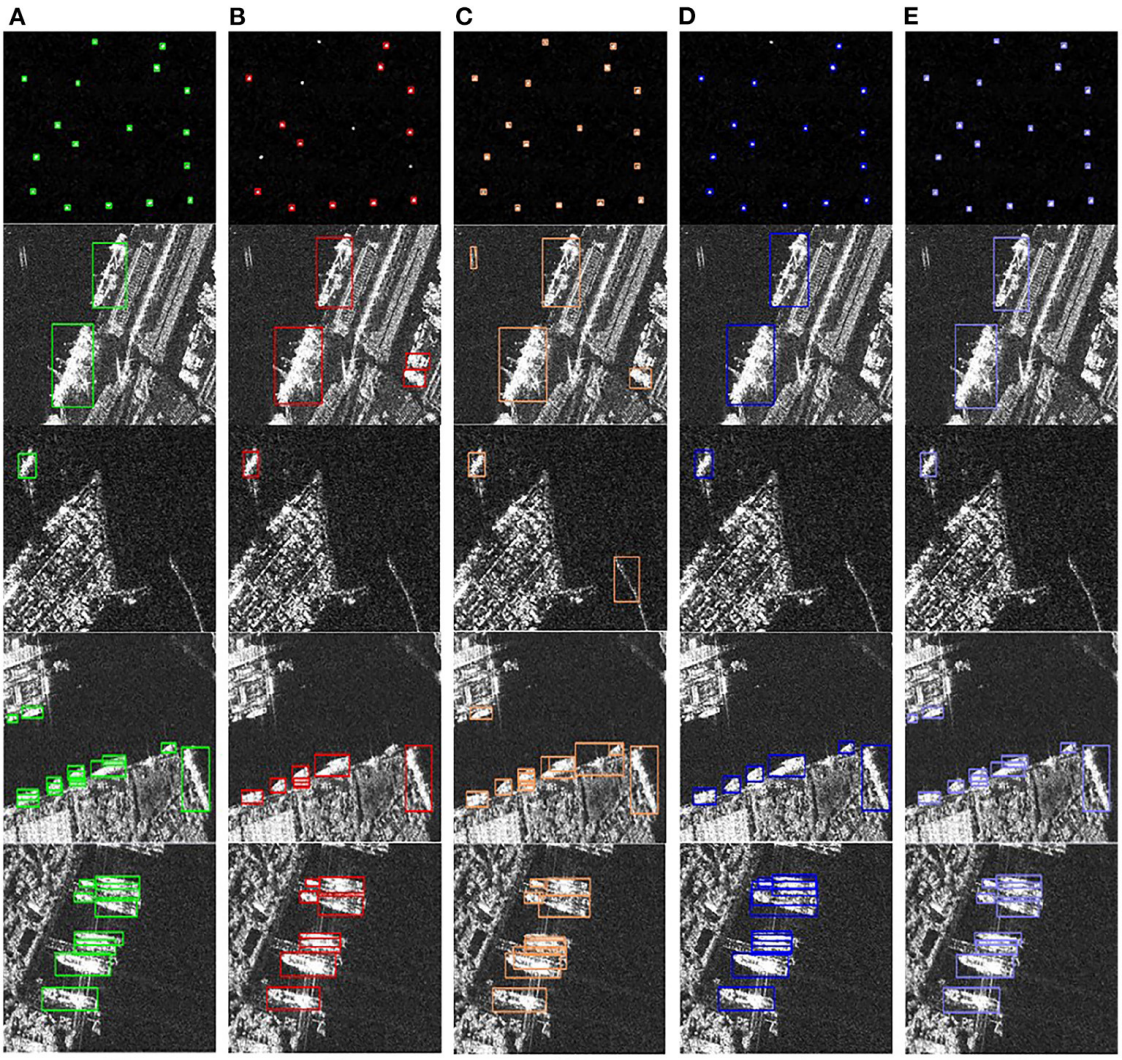
Detection results of different detection methods. **(A)** Ground truth. **(B)** Detection results of Faster R-CNN. **(C)** Detection results of RetinaNet. **(D)** Detection results of VFNet. **(E)** Detection results of the proposed algorithm.

Figure 6 shows the detection results of other target detection methods and the proposed algorithm. In the figure, green indicates the truth box, red indicates the Faster R-CNN detection results, yellow indicates the RetinaNet detection results, blue indicates the VFNet detection results, and purple indicates the detection results of the proposed algorithm. In Figure 6, the first row shows that the proposed algorithm has better detection results for small-scale ships, the second and third rows show that the proposed algorithm can effectively detect targets in complex near-shore scenes, and the fourth and fifth rows show that the proposed algorithm can get better detection results for densely arranged ships, while the other methods have poor detection results. The detection results we obtained show that the proposed algorithm can be better applied to small-scale targets, complex scenes, and densely arranged targets.

## 5. Conclusion

In this article, drawing on the idea that the brain-inspired can effectively use visual targets and their surrounding background information, we propose an improved anchor-free SAR ship detection algorithm based on brain-inspired attention mechanism. The proposed algorithm improves on the anchor-free network, and in order to obtain richer target information, the deep part of the network applies a dense connection module to promote more adequate fusion of deep semantic features, and the shallow part of the network applies a visual attention module to extract features rich in fine-grained details. And in order to enable more accurate target localization in complex scenes, a novel width height prediction constraint is proposed to finally improve the target detection accuracy. After experimental validation, the proposed algorithm achieves better detection results in SAR ship target detection. In addition, there is a shortcoming during the experiment, some densely arranged ships are missed, so we will continue to improve the proposed algorithm in the future, such as considering multimodal information of ship targets, including but not limited to ship target detection under different frequency bands.

## Data availability statement

The datasets analyzed for this study can be found in the online repository. SSDD data can be found here: https://github.com/TianwenZhang0825/Official-SSDD, HRSID data can be found here: https://github.com/chaozhong2010/HRSID.

## Author contributions

CH and HS conceptualized the study. CH wrote the first draft of the manuscript and performed the experiments. CH, HS, and YW performed data analysis. JL and LC collected and analyzed the data. HS, YW, JL, and LC revised the manuscript. All authors contributed to the article and approved the submitted version.

## Funding

This work was supported in National Natural Science Foundation of China: 62101041.

## Conflict of interest

The authors declare that the research was conducted in the absence of any commercial or financial relationships that could be construed as a potential conflict of interest.

## Publisher's note

All claims expressed in this article are solely those of the authors and do not necessarily represent those of their affiliated organizations, or those of the publisher, the editors and the reviewers. Any product that may be evaluated in this article, or claim that may be made by its manufacturer, is not guaranteed or endorsed by the publisher.
